# Carbohydrate utilization by the gut microbiome determines host health responsiveness to whole grain type and processing methods

**DOI:** 10.1080/19490976.2022.2126275

**Published:** 2022-09-21

**Authors:** Caroline Smith, Mallory J. Van Haute, Yibo Xian, Rafael R. Segura Munoz, Sujun Liu, Robert J. Schmaltz, Amanda E. Ramer-Tait, Devin J. Rose

**Affiliations:** a Department of Food Science and Technology, University of Nebraska-Lincoln, Lincoln, NE, USA; b Grain Research and Quality, Ardent Mills, Denver, CO, USA; c Nebraska Food for Health Center, University of Nebraska-Lincoln, Lincoln, NE, USA; d Research and Development, Synbiotic Health, Lincoln, NE, USA; e Department of Systems & Synthetic Microbiology, Max Planck Institute for Terrestrial Microbiology, Marburg, Germany; f Department of Agronomy and Horticulture, University of Nebraska-Lincoln, Lincoln, NE, USA

**Keywords:** Gut microbiome, extrusion, fermentable carbohydrates, whole wheat, brown rice

## Abstract

Little is known about how interactions among grain processing, grain type, and carbohydrate utilization (CU) by the microbiome influence the health benefits of whole grains. Therefore, two whole grains – brown rice and whole wheat – and two processing methods – boiling (porridge) and extrusion – were studied for their effects on host metabolic outcomes in mice harboring human microbiomes previously shown *in vitro* to have high or low CU. Mice carrying either microbiome experienced increases in body weight and glycemia when consuming Western diets supplemented with extruded grains versus porridge. However, mice with the high but not low CU microbiome also gained more weight and fat over time and were less glucose tolerant when consuming extruded grain diets. In high CU microbiome mice, the exacerbated negative health outcomes associated with extrusion were related to altered abundances of *Lachnospiraceae* and *Ruminococcaceae* as well as elevated sugar degradation and colonic acetate production. The amplicon sequence variants (ASVs) associated with extruded and porridge diets in this *in vivo* study were not the same as those identified in our prior *in vitro* study; however, the predicted functions were highly correlated. In conclusion, mice harboring both high and low CU microbiomes responded to the whole grain diets similarly, except the high CU microbiome mice exhibited exacerbated effects due to excessive acetate production, indicating that CU by the microbiome is linked to host metabolic health outcomes. Our work demonstrates that a greater understanding of food processing effects on the microbiome is necessary for developing foods that promote rather than diminish host health.

**Abbreviations**: CU- carbohydrate utilization; SCFA- short-chain fatty acids; GF- germ-free; HMA, human-microbiome associated; ipGTT- intraperitoneal glucose tolerance test; HOMA-IR- Homeostatic Model Assessment for Insulin Resistance; AUC- area under the glycemia curve; ASV- amplicon sequence variant; lf- low-fat; wd- Western diet; wd_wwp- Western diet containing whole wheat porridge; wd_wwe- Western diet containing whole wheat extrudate; wd_bre- Western diet containing brown rice extrudate; wd_extr- Western diet containing either whole wheat or brown rice extrudate.

## Introduction

Whole grain intake is associated with a reduced risk of type 2 diabetes, cardiovascular disease, cancer, and obesity.^1^ Moreover, whole grains and dietary fibers also influence the composition and function of the gut microbiota.^2,3^ Although the effects on specific microbial taxa are varied, whole grains generally increase microbial diversity, particularly in subjects who habitually consume diets low in fiber or whole grains.^3^ Unfortunately, most Westerners do not consume adequate levels of dietary fiber and whole grains despite decades of recommendations and public health initiatives promoting such behavior.^4^ In the United States, 94% of adults fail to consume adequate levels of dietary fiber.^5^ Adult intake of whole grains also falls far below published recommendations (3 ounce equivalents/2000 kcal recommended versus <1 ounce equivalents/2000 kcal actual consumption) despite consumption of sufficient amounts of grain products (6 ounce equivalents/2000 kcal).^6^ Consequently, efforts to maximize the benefits of dietary fiber are critical because most Americans do not get enough of this vital nutrient.

Processing holds promise as a way to overcome fiber consumption challenges by manipulating the interactions between dietary carbohydrates and the microbiota to improve human health.^7^ For example, cooking high starch foods has been shown to improve gut bacterial physiology by controlling starch availability.^8^ However, among high starch foods processed by different cooking methods, the outcomes of processing can be unpredictable. In a previous *in vitro* study using human gut microbiota, we expected that extrusion, a severe processing operation that uses pressure, mechanical shear, and higher temperatures (>120°C) to process grains into foods such as breakfast cereals and crispy snacks, would increase carbohydrate utilization (CU) by the microbiome and lead to positive changes in microbiota composition and functionality compared with less severe processing methods such as boiling.^9^ Although extrusion increased non-digestible CU, it also changed fermentation dynamics such that fast-growing bacteria, including *Acinetobacter, Enterococcus*, and *Staphylococcaceae*, outcompeted beneficial butyrate-producing *Ruminococcaceae* and *Lachnospiraceae*. In contrast, less severe processing methods (i.e., boiling and sourdough breadmaking) lowered total carbohydrate utilization compared to extrusion but increased butyrate production and the abundance of butyrate-producing *Ruminococcaceae* and *Lachnospiraceae*. Notably, only microbiomes capable of metabolizing a high proportion of the non-digestible carbohydrates (i.e., high CU) changed their fermentation patterns in response to processing method. Microbiomes with low CU showed no significant differences among extrusion, boiling, sourdough breadmaking or any other processing operation. These key findings provide an important potential explanation for why some individuals “respond” to dietary interventions aimed at improving human health through their interactions with the gut microbiome while others do not.^10,11^

Although previous studies have demonstrated that processing, grain type, and the microbiome each individually influence the health benefits experienced by those who consume whole grains,^1,12–14^ we do not yet understand how these factors interact with one another to alter host health outcomes in a predictable fashion. Indeed, results from our prior work suggest that only individuals who harbor high CU microbiomes are able to experience the potential benefits or disadvantages of processed whole grain treatments. Therefore, the purpose of this study was to examine the combinatorial effects of whole grain type and processing method on host health using mice harboring either high or low CU microbiomes. Two whole grains, brown rice and whole wheat, and two processing methods, boiling and extrusion, were studied for their effects on the microbiome and host metabolic outcomes. We hypothesized that mice harboring a high CU microbiome would experience metabolic improvements when fed boiled whole grains compared to extruded whole grains. In contrast, no appreciable metabolic differences would be noted for mice harboring a low CU microbiome when fed either extruded or boiled whole grains. We further hypothesized that the metabolic effects experienced by the high CU microbiome mice would be exaggerated when consuming whole wheat diets compared to brown rice diets due to the higher concentration of non-digestible carbohydrates in wheat versus brown rice.^15^

## Materials and methods

### Whole grain processing

Hard red wheat was obtained from Bay State Milling (Quincy, MA, USA). Wheat kernels were dried at 40°C for 16 h before milling according to Doblado-Maldonado et al.^16^ All milled fractions obtained from the mill were mixed together to obtain whole wheat flour. Brown rice flour was obtained from Bay State Milling (Quincy, MA, USA).

To make the whole wheat porridge, water (530 g) was brought to a boil on a gas range. Once boiling, 1 g of salt and 100 g whole wheat flour were added and the mixture was cooked for 5 min under rapid manual stirring (~60 revolutions per min) at 85–95°C. This process was repeated for 30 batches. The boiled wheat porridge was then cooled to room temperature before being frozen at −80°C and then freeze dried. The freeze died product was then coarsely ground and then milled with a pin mill (Buhler, Uzwil, Switzerland) twice to obtain a fine powder. All batches of freeze dried, milled whole wheat porridge were mixed together for incorporation into the mouse feed.

For extrusion, samples were processed as described previously.^17^ One kilogram of flour (whole wheat or brown rice) was mixed in a stand mixer (c-100, Hobart, Troy, OH) for 10 min with 1% salt (w/w) and water to adjust the moisture content to 20% (dry weight basis). The mixtures were equilibrated in closed containers at 4°C overnight. The moisture-adjusted whole grain flours were then extruded using a twin-screw extruder equipped with a single stage mixing zone and a 3 mm outlet die at 250 rpm, 3:1 compression ratio, and a 20:1 L/D ratio (CW Brabender Instruments, NJ, USA). The extruder was operated by a direct current drive unit (Intelli-Torque, Pastic Corder Lab-station, C.W. Brabender) with a 5.6 kW motor. The flour was fed into the extruder using a volumetric feeder (FW 40 Plus, C. W. Brabender) set at a constant flow rate of ~50 g/min. Barrel temperatures were set at 60°C (zone 1; inlet), 70°C (zone 2), 120°C (zone 3), and 120°C (zone 4; die assembly). Samples were collected from the extruder die once steady state had been reached. Extrudates were then dried in a convection oven overnight at 70°C to complete the extrusion processing. Although the product contained very low moisture at this stage (~3%), the dried extrudates were subject to freeze drying (3600, Freeze Dry Co., Pine River, MN) because the whole wheat porridge was freeze dried following processing. The freeze-dried material was coarsely ground and then milled with a pin mill (Buhler, Uzwil, Switzerland) twice to obtain a fine powder.

Composition of the whole wheat porridge and whole grain extrudates were measured on the processed flours. Moisture, protein, lipid, dietary fiber, and starch were measured using standard methods.^18–22^ Degree of starch gelatinization was measured using an enzymatic method.^23^ Sugars (glucose, fructose, and sucrose) were measured as previously described with some modifications.^24^ Sample (150 mg) was dispersed in 1.5 mL of water and stirred for 1 h at room temperature. The slurry was then centrifuged at 4000 *g* for 10 min and the supernatant was filtered through a 0.45 μm syringe filter before injecting into an HPLC (1260, Agilent, Santa Clara, CA, USA) equipped with an HPX-87P carbohydrate analysis column (300 × 7.8 mm, Bio-Rad, Hercules, CA, USA) with an ionic form H^+^/CO_3_^−^ deashing guard column. Results were compared to authentic glucose, fructose, and sucrose standards (0–1 mg/mL) to calculate the concentration in the processed grain samples.

### Selection of high CU and low CU microbiomes

We previously identified groups of “high CU” and “low CU” microbiomes based on contrasting percentages of carbohydrates fermented during *in vitro* fermentation.^9^ In that study, ten microbiomes were subjected to *in vitro* fermentation using whole wheat processed by five different methods and CU was quantified by high-performance anion exchange chromatography. These ten microbiomes separated into two groups based on CU (31.1 ± 1.1% versus 19.3 ± 1.2%, P < .001), where CU was defined as the percentage of carbohydrates fermented during 12 h of *in vitro* fermentation.

For this study, the microbiome that was closest to the mean CU for each group was selected for inoculation into mice in this study (donor 1 for the high CU microbiome and donor 2 for the low CU microbiome). The same fecal samples collected from the previous study were used in this study. Two stool samples were collected from each donor (<2 weeks apart) and blended (model 2774 blender; Sunbeam) separately with sterile phosphate-buffered saline, pH 7.0 (1:9 wt/vol) containing 10% glycerol as a cryoprotectant for 1 min. The slurry was then filtered through four layers of sterile cheesecloth and frozen at −80°C. The two fecal slurries from each donor were defrosted and pooled on the day the mice were inoculated and stored on ice until use. All procedures involving human subjects were approved by the Institutional Review Board of the University of Nebraska–Lincoln (20160816311EP).

### Experimental diets

The diets used in the study were prepared and irradaited by Research Diets (New Brunswick, NJ USA) (Table S2). The control diet was a defined rodent diet meant to represent a human Western diet in terms of macronutrient caloric distribution (D12451). A low fat diet (D12450K) was included to confirm metabolic aberrations induced by the Western diet. The processed whole grain samples were incorporated into the mouse diets at 18.5% of the total diet. The casein, corn starch, maltodextrin, soybean oil, and cellulose were reduced in the grain-containing samples compared with the control Western diet to keep the macronutrient composition consistent for total protein, starch, fat, and dietary fiber among each diet. Because of the differences in dietary fiber concentration among processed whole grain samples, the fiber from the grain treatment in each diet varied from as little as 0.3% to 1.8% of the whole diet. The level of whole grains included in the treatment diets were approximated to match the recommendations in the USDA Dietary Guidelines for Americans^6^ These Guidelines recommend 3 servings (ounce equivalents) per day for a standard 2000 kcal diet. Assuming 1 serving of whole grain contributes about 120 kcal to the diet, then 3 servings of whole grain would comprise 370 kcal, or about 18.5%, of an overall diet.

### Human microbiota-associated mice

The Institutional Animal Care and Use Committee at the University of Nebraska–Lincoln approved all procedures involving animals (Project ID: 1700). The study was conducted in two arms (one for each human microbiome) due to the number of mice on study. Germ-free (GF) C57BL/6 male mice were bred and reared in flexible film isolators and maintained under gnotobiotic conditions (including temperature 20°C, relative humidity 60%, 14 h light/10 h dark cycle) by the Nebraska Gnotobiotic Mouse Program at the University of Nebraska–Lincoln. Germ-free status of the breeding colony was checked routinely as previously described.^25^ At seven weeks of age (±11 d), GF mice were transferred from isolators to autoclaved individually ventilated cages mounted on racks with positive airflow as previously described,^25^ colonized immediately with either the high CU or low CU human stool microbiome, and housed two to three mice per cage. Cages were only opened in a biosafety cabinet. To establish human microbiome-associated (HMA) mice, 100 μL of each human fecal slurry was orally gavaged into mice once. After receiving a microbiome, mice were fed an autoclaved chow diet (LabDiet 5K67, Purina Foods, St. Louis, MO) for two weeks and allowed to acclimate prior to beginning the study. After two weeks, HMA mice were randomly assigned to one of the five dietary treatments (n = 10–11 mice/treatment) and then fed irradiated test diets for 12 weeks. No significant differences in body weight were observed among treatment groups on the day of randomization (data not shown). Diet replacement and recording of both food intake and body weights were performed weekly. Body composition was measured bi-weekly (Minispec LF50, Bruker, Billerica, MA).

Feces were collected from individual mice three times during the experiment (weeks 0, 1, and 11) and stored at −80°C until further analysis. After 12 weeks of feeding experimental diets, mice were euthanized via CO_2_ asphyxiation. Blood was harvested by cardiac puncture and plasma was collected by centrifugation at 13,000 × *g* for 3 min at 4°C. Cecal and colonic tissues and contents were weighed at the time of collection. All biological samples were snap frozen in liquid nitrogen and stored at −80°C until analysis.

### Intraperitoneal glucose tolerance test

An intraperitoneal glucose tolerance test (ipGTT) was performed after 11 weeks of dietary intervention.^26^ Mice were fasted in clean cages for 6 h prior to the test. Thirty minutes prior to the test, blood was collected from the tip of the tail to measure fasting glucose concentration using a glucose meter (ACCU-CHEK, Aviva Plus system, Indianapolis, IN, USA). An aliquot of blood was also saved for subsequent insulin analysis via ELISA (Mercodia Insulin ELISA Kit, Uppsala Sweden). At time zero, a glucose solution (20 g/100 mL) was injected into the peritoneal cavity (1 g glucose/kg body weight). Blood glucose levels were measured using a glucose meter (Roche Diagnostics, Indianapolis, IN) at 0, 30, 60, 90, 120, and 150 min after glucose injection. An index of insulin resistance [Homeostatic Model Assessment for Insulin Resistance (HOMA-IR)] was calculated using the formula: [fasting glucose (mg/dL) * fasting insulin (μU/mL)]/405.^27^

### Cecal and colon short-chain fatty acids

SCFA were extracted from the cecum and colon collected at necropsy. SCFA were measured by gas chromatography as described^28^ with 7 mM 2-ethylbutyric acid as the internal standard. SCFA were reported as the total quantity of SCFA in each tissue by multiplying the concentration by the weight of tissue contents.

### Characterization of the fecal microbiota composition

The fecal samples were analyzed for differences in the microbiome using 16S rRNA gene sequencing. A kit from Biovet (BioSprint 96 One-For-All Vet Kit, Quebec Canada) was used for DNA extraction. Microbiome characterization was performed by amplicon sequencing of the V4 region of the 16S rRNA gene on the Illumina MiSeq platform using the MiSeq Reagent kit v2 (2 × 250 bp) following the protocol of Kozich et al.^29^ Sequences were demultiplexed and barcodes were removed prior to sequence analysis with the QIIME 2 platform.^30^ Sequencing reads are available at: PRJNA690589. Sequence quality control, trimming and denoising was performed with DADA2.^31^ Forward and reverse reads were trimmed to maintain sequence qualities above a phred score of 30. Using DADA2, sequences were dereplicated into 100% amplicon sequence variants (ASVs) for exact sequence matching. Taxonomy was assigned using the SILVA database.^32^ Reads were rarefied to a sampling depth of 7,662 prior to analysis. Prediction of metagenome function of ASVs was performed using pathway analysis in PiCRUSt2^33^ using the stratified and per-sequence contribution option to determine the predicted pathway abundance contributed by each individual sequence. Pathways were then grouped into hierarchical classifications as found in https://metacyc.org/.

### Statistical analysis

For analysis of host parameters, a one-way analysis of variance (ANOVA) was performed (version 9.4, SAS Institute, Bray, NC USA). Body weights while feeding test diets and blood glucose concentrations during the ipGTT were analyzed using a repeated measures ANOVA with dietary treatment and time and their interaction as the factors and mouse as the experimental unit on which repeated measures were taken. When the ANOVA F-value was significant (p < .05), Tukey’s honestly significant difference (HSD) was used to compute differences among treatments. Because the study was conducted in two arms (one for each human microbiome), statistics were run by microbiome.

Fecal microbiota analyses were conducted using R (version 4.0.2) with various packages as indicated, except for linear discriminate analysis effect size (LEfSe) analysis,^34^ which was performed using the online Galaxy module (http://huttenhower.sph.harvard.edu/galaxy). Diversity of each fermented and fecal sample was determined using the phyloseq package.^35^ For β-diversity, unweighted and weighted UniFrac distances were calculated and then visualized using principal coordinates analysis. Homogeneity of group variances was performed using the vegan package.^36^ For α-diversity, observed ASVs and Shannon diversity were calculated and differences among treatments were analyzed using a non-parametric Kruskal–Wallis test followed by a Dunn test using the rstatix package.^37^ For further analysis, low abundance ASVs that occurred at <0.1% in all samples were considered possible spurious sequences and were filtered out, resulting in 455 ASVs across both microbiomes, with 319 ASVs in the high CU microbiome mice and 212 ASVs in the low CU microbiomes (76 shared ASVs). To analyze shifts in individual ASVs over time, the DESeq2 package was used^38^ with time as the factor in the model (week 11 vs week 0); analyses were grouped by microbiome and dietary treatment.

To determine ASVs that were significantly associated with extruded diets versus the porridge diet after 11 weeks of feeding, linear discriminate analysis effect size (LEfSe) was used using data at 11 weeks. For correlation analysis between host parameters and abundance of different ASVs, Spearman correlations were calculated. Results from the DESeq2, correlation, and pathway contribution analyses were visualized using the ComplexHeatmap package.^39^ Dietary treatments were clustered using the complete-linkage method based on Euclidean distance. ASVs were clustered using ggtree.^40^ All other data were plotted using the ggplot2 package.^41^ Multipanel figures were arranged using the cowplot package.^42^

## Results

### *In vitro* fermentation of processed whole grains identified high and low carbohydrate utilizing microbiomes

**Figure 1.**
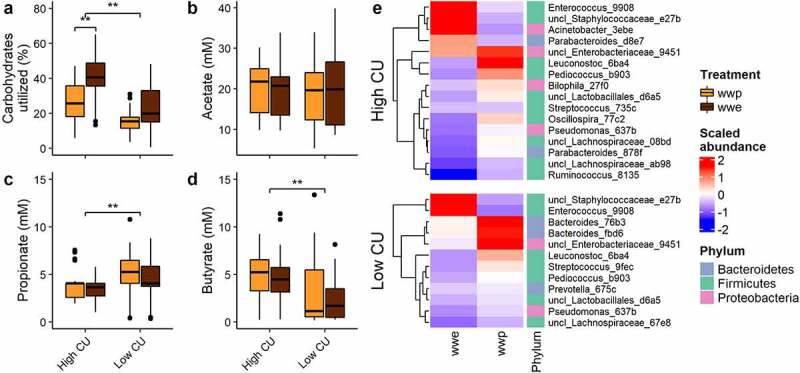
***In vitro* fermentation of processed whole grains identified high and low carbohydrate utilizing microbiomes from donors**. (a) Carbohydrates utilized; (b-d) short-chain fatty acid production; (e) differentially abundant ASVs. ASVs were named by genus (or lowest taxonomic rank available) followed by the first four characters of the feature ID generated from QIIME 2; wwp, whole wheat porridge; wwe, whole wheat extrudate; **p < .01; data from.^9^

In a previous *in vitro* study examining the effects of whole-grain processing on gut microbiota composition and function,^9^ extrusion, a severe processing method, negatively affected microbiome composition and function compared to other milder processing methods, but only in microbiomes demonstrating a high carbohydrate utilization (CU) pattern. Specifically, extrusion resulted in greater CU than boiling (porridge) for the high CU microbiomes (Figure 1a), but also increased ASVs from *Acinetobacter, Enterococcus*, and *Staphylococcaceae* at the expense of key cell wall degrading bacteria from the *Ruminococcaceae* and *Lachnospiraceae* families (Figure 1e). High CU microbiomes also produced significantly more butyrate than low CU microbiomes, which were more propiogenic (Figure 1b-d).

### Whole grain porridge and extrudates showed differences in composition

The composition of the whole wheat porridge and whole grain extrudates was as expected, with a very low concentration of dietary fiber in the brown rice extrudate (1.69%) compared with the whole wheat extrudate (8.75%; Table S1). The extrusion processing diminished the total fiber content compared with the porridge sample (8.75% versus 9.70%). There were marked differences in degree of starch gelatinization among the samples, with the extruded diets containing a much greater proportion of gelatinized starch (whole wheat extrudate: 95.7%; brown rice extrudate: 88.6%) compared with the whole wheat porridge (68.3%). The whole wheat porridge and the whole wheat extrudate represented the contrast between a minimally processed grain and a severely processed grain, respectively. The whole wheat extrudate and the brown rice extrudate represented the contrast between different grain types when processed using the same methodology.

### Extruded diets worsened host metabolic health compared with non-extruded diets in mice harboring a high CU microbiome

Given the differential microbiome and SCFA responses to processed whole grains by the high CU microbiomes versus the low CU microbiomes *in vitro*, we examined whether these relationships persisted *in vivo*. Specifically, we hypothesized that mice fed a diet containing whole grain porridge would experience enhanced metabolic benefits compared to mice fed a diet containing extruded whole grain, but only in hosts harboring a high CU microbiome.

To test this hypothesis, we inoculated germ-free mice with high CU and low CU microbiomes.^9^ The high CU microbiome had more observed ASVs, but its Shannon diversity was similar to the low CU microbiome (Fig. S1), indicating that although species richness was greater for the high CU microbiome, species distrubution was uneven. The high CU microbiome had a greater abundance of *Verrucomicrobia*, while the low CU was higher in *Actinobacteria* (Fig. S1). In addition, the high CU microbiome had greater levels of *Prevotella* and more *Ruminococcaceae*, especially *Ruminococcus*, compared to the low CU microbiome. The composition of the *Lachnospiraceae* family was also different between the two microbiomes, with more *Roseburia, Coprococcus*, and [*Eubacterium*] *xylanophilium* group present in the high CU microbiome and more *Fusicatenibacter, Dorea, Blautia, Anaerostipes*, and [*Eubacterium*] *hallii* group present in the low CU microbiome. These differences were similar to those previously reported for the whole high and low CU microbiome groups.^9^

**Figure 2.**
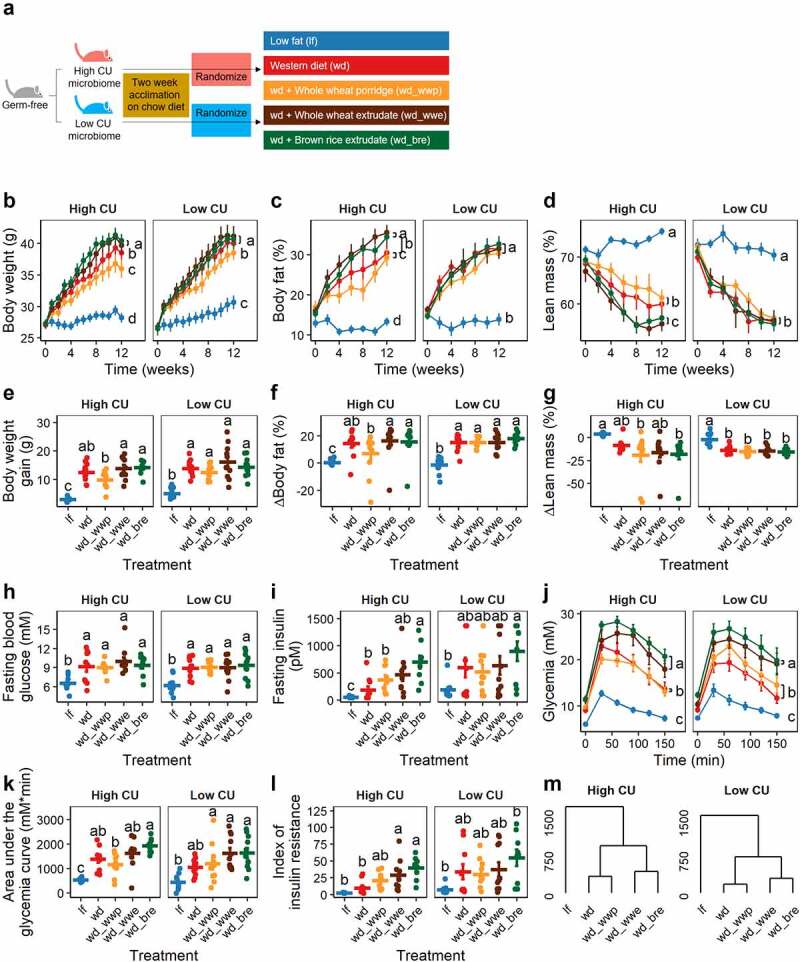
**Extruded diets worsened host metabolic health compared with non-extruded diets in mice harboring a high CU microbiome**. Study design (a); body weight (b), body fat (c), lean mass (d), body weight gain (e), change in body fat (f), change in lean mass (g), fasting blood glucose (h), fasting insulinemia (i), glycemia during intraperitoneal glucose tolerance test (j), area under the glycemia curve (k); index of insulin resistance (l), complete linkage clustering based on Euclidean distance of treatment means of data in panels B-L (m); lf, low fat control; wd, Western diet; wd_wwp, wd + whole wheat porridge; wd_wwe, wd + whole wheat extrudate; wd_bre, wd + brown rice extrudate; treatments marked with different letters within panel are significantly different from one another (Tukey’s HSD p < .05).

These two microbiomes were used to create HMA mice harboring either a high CU or low CU human stool microbiome. After a two-week acclimation period, HMA mice were fed one of five test diets containing processed whole grains for 12 weeks (Figure 2a). The diets included a control low-fat diet, a control Western diet, and three energy and macronutrient-matched Western diets supplemented with either boiled whole wheat (whole wheat porridge), extruded whole wheat, or extruded brown rice. The metabolic health of the mice was assessed by measuring changes in body weight, body composition, and glucose tolerance.

All mice receiving a Western diet gained significantly more body weight and fat, experienced elevated blood glucose and insulin levels, and were more insulin resistant following a glucose challenge compared to mice fed a low fat control diet, all of which are features associated with the development of metabolic syndrome (Figure 2b-l). No significant differences in feed intake were observed among mice harboring either microbiome for any of the test diets (high CU, 19.6 ± 1.5 g/week, p = .59; low CU, 22.6 ± 2.7 g/week, p = .18).

For mice inoclulated with the high CU microbiome, body weight gain and fat percentage were significantly higher when consuming a Western diet supplemented with either extruded grain compared to mice receving the whole wheat porridge diet. Weight gain and fat accretion for mice fed the control Western diet were between results obtained for mice receiving a Western diet supplemented with whole grain treatments (Figure 2b-g). Mice consuming the brown rice extrudate experienced higher fasting insulin levels and were more insulin resistant following a glucose challenge compared with mice consuming whole wheat porridge (Figure 2i-l). Insulin levels and glycemia profiles for mice consuming whole wheat extrudate were intermediate to profiles for mice receiving the other two whole grain diets. Glycemia was also lower for mice consuming the control Western diet or a Western diet supplemented with whole wheat porridge compared to mice consuming the extruded whole grain diets (Figure 2j).

In contrast to the findings for mice carrying a high CU microbiome, mice inoculated with the low CU microbiome experienced far fewer differences in metabolic features among whole grain dietary treatments. A significant decrease in body weight over time was seen in mice consuming the whole wheat porrige diet compared with the other whole grain diets (Figure 2b), but no differences were observed in body weight gain or body composition profiles (Figure 2c-g). Mice consuming the whole wheat porridge diet had lower glycemia during a glucose challenge compared with mice receiving the extruded diets (Figure 2j). However, no other differences in metabolic parameters were observed among mice carrying the low CU microbiome and consuming whole grains (Figure 2h, i, k, l).

Although a greater number of significant differences among dietary treatments for host metabolic parameters were observed for mice harboring the high CU microbiome, mice receiving either microbiome showed similar clustering of treatment means (Figure 2m). In general, mice consuming either extruded diet (whole wheat or brown rice) were less metabolically healthy than mice consuming the other diets. Altogether, these results demonstrate that the host metabolic response to extruded diets was worse than the porridge diet in mice regardless of microbiome, but the negative effects were greatly exacerbated in the mice harboring the high CU microbiome compared to the low CU microbiome.

### Mice inoculated with the high CU microbiome showed greater variance in microbiota composition among treatments during feeding compared with the low CU microbiome mice

**Figure 3.**
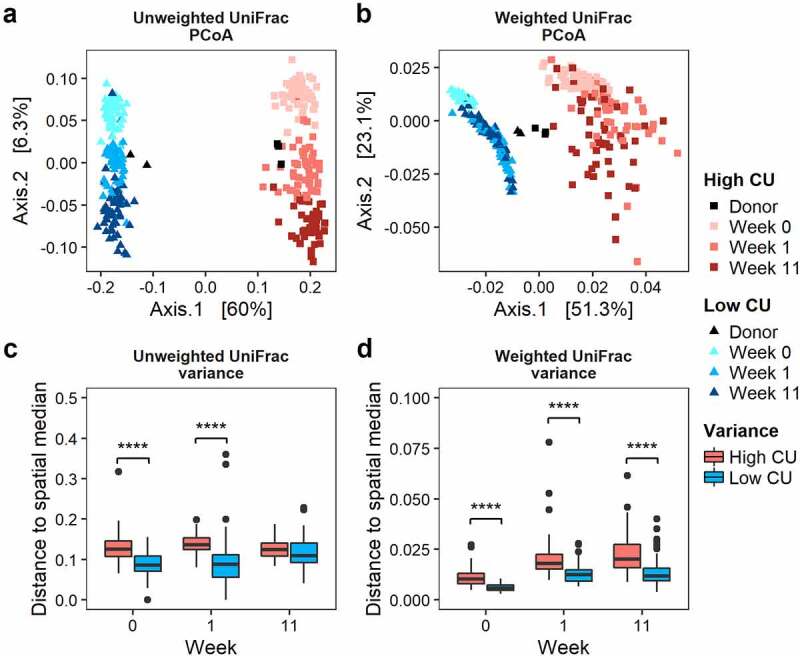
**Mice inoculated with the high CU microbiome showed greater variance in microbiota composition among treatments during feeding compared with the low CU microbiome mice**. Principal coordinate analysis biplots of unweighted (a) and weighted (b) UniFrac distance matrices and group dispersions (variances) based on unweighted (c) and weighted (d) UniFrac distances; ****p < .001 (t-test).

Because the high CU microbiome mice experienced a greater number of significant metabolic differences among dietary treatments compared to the low CU microbiome mice, we expected that the high CU microbiome mice would show greater variation in microbiome composition among treatments. Upon transfer into mice, both fecal microbiomes exhibited compositional shifts based on fecal collection week (Figure 3a, b). During the two-week acclimation period, the shift in microbiota composition from the donor inocula ranged from 0.027 to 0.11 based on unweighted UniFrac distance, which was substantially smaller than the difference between the high and low CU fecal inocula (0.27 ± 0.01) with no significant difference between high and low CU microbiome mice (Figure 3c). In contrast, the change in microbiota composition after inoculation and acclimation based on weighted UniFrac distance ranged from 0.021 to 0.033, which was larger than the difference between the high and low CU fecal inocula (0.0087 ± 0.0015) with a slight but significantly greater shift for the high CU microbiome mice compared with the low CU microbiome mice (Figure 3d). Together, these observations indicate that the minor alterations in microbiome composition during the acclimation period were mostly changes in abundance rather than loss/gain of taxa. After 11 weeks of feeding, the variance in β-diversity among treatments was significantly greater for the high CU microbiome mice compared with the low CU microbiome mice for weighted UniFrac distance but not for unweighted UniFrac distance (Figure 3c, d). These observations suggest that the high CU microbiome mice exhibited a more dramatic response to treatment conditions compared with the low CU microbiome mice and that differences among dietary treatments may have been more dependent on shifts in abundance of certain species rather than simply presence or absence.

For α-diversity, very few differences in observed ASVs or Shannon diversity were observed among treatments for either microbiome with only the low CU microbiome mice showing any signifciant differences at all (Fig. S2). However, over 11 weeks of feeding, there was a general trend for increasing α-diversity during the study. This general trend was more pronounced for mice receiving the high CU microbiome and was more evident for observed ASVs than for Shannon diversity. These results suggest that the dietary treatments had more of an effect at the ASV level, even among the low abundance ASVs, rather than affecting the evenness of the community, especially in the high CU microbiome mice.

### Changes in microbiome composition during feeding clustered by processing method in mice harboring the high CU microbiome

**Figure 4.**
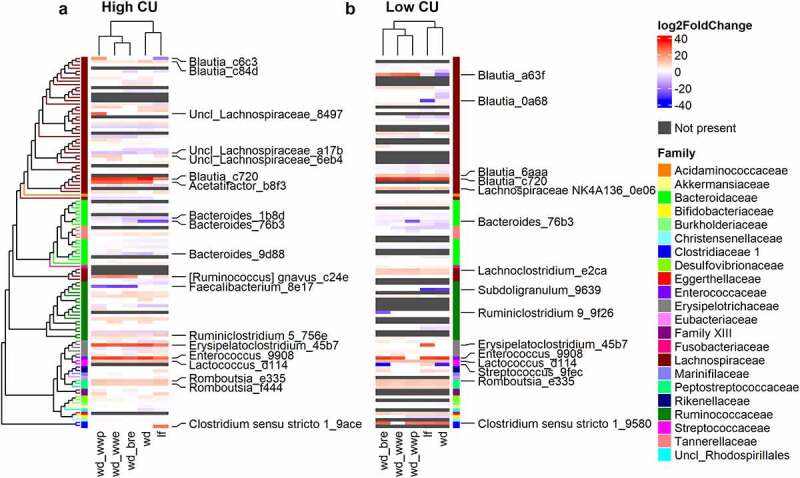
**Changes in microbiome composition during feeding clustered by processing method in mice harboring the high CU microbiome**. ASVs with significant log_2_ fold changes (DESeq2 padj<0.05) during feeding of high (a) and low (b) CU microbiomes; ASVs are clustered by phylogeny; treatments are clustered by the complete-linkage method based on Euclidean distance; ASVs with the largest absolute value of the log_2_ fold change (top 20%) are labeled; lf, low fat control; wd, Western diet; wd_wwp, wd + whole wheat porridge; wd_wwe, wd + whole wheat extrudate; wd_bre, wd + brown rice extrudate.

The β-diversity results suggested that shifts in microbal abundances – rather than the presence or absence of certain species – may explain the enhanced differences among dietary treatments in the high CU microbiome mice. Thus, we analyzed shifts in microbiome composition at the ASV level from week 0 to week 11 for each treatment and microbiome (Figure 4). In both microbiomes, 30% of the ASVs showed significant changes during feeding in at least one dietary treatment condition. Since the high CU microbiome mice had more observed ASVs, this result translated into 82 ASVs with significant shifts over time in the high CU microbiome mice compared to 61 for the low CU microbiome mice. While the specific ASVs that changed in each microbiome were different, remarkably similar shifts were evident when the log_2_ fold changes during feeding were clustered by phylogeny. Notably, closely related ASVs exhibited similar shifts during feeding in either microbiome. For example, different ASVs from *Blautia, Erysipelactoclostridium, Enterococcus*, and *Romboutsia* tended to increase, while ASVs from *Bacteroides* tended to decrease.

More revealing than the shifts in individual ASVs during feeding were the clustering patterns observed among the dietary treatments for each microbiome. In mice inoculated with the high CU microbiome, the extruded diet treatments clustered separately from the other diets (Figure 4a). This result is similar to the clustering based on host metabolic outcomes, where extruded diets also clustering separately from other dietary treatments (Figure 2m). In contrast, shifts in microbiome composition clustered by grain type rather than processing method for mice inoculated with the low CU microbiome (Figure 4b), which was different from the clustering of metabolic outcomes (Figure 2m). Together, these findings suggest that the exacerbated negative host metabolic outcomes in high CU microbiome mice fed the extruded diets were related to changes in microbiota composition.

### Extruded diets resulted in differential ASVs with strong correlations to negative host outcomes in the high CU microbiome mice

**Figure 5.**
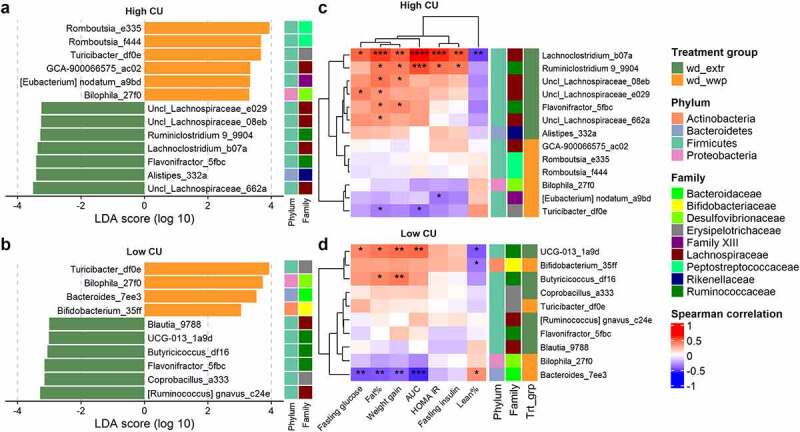
**Extruded diets resulted in differential ASVs with strong correlations to negative host outcomes in the high CU microbiome mice**. Differential ASVs associated with extruded (wd_extr) and non-extruded (wd_wwp) diets in high CU (a) and low CU (b) microbiomes; correlations between differential ASVs in high CU (c) and low CU (d) microbiomes and host metabolic variables; wd_extr, Western diet + whole wheat extrudate (wd_wwe) and brown rice extrudate (wd_bre); non-extruded diet included the Western diet + whole wheat porridge (wd_wwp); ASVs were named by genus (or lowest taxonomic rank available) followed by the first 4 characters of the feature ID generated from QIIME2; *p < .05, **p < .01, ***p < .001, ****p < .0001 (Spearman correlation with Benjamini-Hochberg p-value adjustment).

Because high CU microbiome mice consuming the extruded diets clustered together for both host metabolic outcomes and shifts in microbiome composition during feeding, we considered that processing method (extruded versus not extruded) was more important for shaping metabolic health and microbiome composition than grain type (whole wheat versus brown rice). We therefore examined differentially abundant ASVs from mice consuming either extruded diet (whole wheat or brown rice) compared with the non-extruded whole grain diet (porridge). There were seven ASVs that were significantly associated with the extruded diets and six ASVs that were associated with the non-extruded (porridge) diet (Figure 5a). We also examined differentially abundant ASVs between extruded versus non-extruded diets for the low CU microbiome mice for comparison. In this background, six ASVs were increased on the extruded diets and four ASVs increased on the non-extruded diet (Figure 5b). Although these ASVs were diverse (from Actinobacteria, Bacteroidetes, Firmicutes, and Proteobacteria), most ASVs associated with extrusion were from the *Lachnospiraceae* or *Ruminococcaceae* families (6/7 and 5/6 for the high and low CU microbiome mice, respectively). One ASV was associated with extrusion in both microbiomes (Flavonifractor_5fbc), and two ASVs were significantly associated with the non-extruded diet in both microbiomes (Bilophila_27f0 and Turicibacter_df0e).

To examine the relationship between these ASVs and host health, we correlated the abundances of these taxa at week 11 with host metabolic phenotypes. For the high CU microbiome mice, correlations with host metabolic parameters clustered by dietary treatment (Figure 5c). Specifically, ASVs associated with feeding extruded diets were strongly and positively correlated with poor host health [i.e., 18 significant positive correlations to increased body weight/body fat or poor glucose metabolism ranging from ρ(0.33, 0.62); adjusted p(0.04, 3.4 × 10^−5^)], while ASVs associated with non-extruded diets were moderately negatively correlated with poor host health [i.e., three significant negative correlations ranging from ρ(−0.40, −0.35); adjusted p(0.02, 0.04)]. In contrast, correlations with host metabolic parameters in the low CU microbiome mice did not cluster by dietary treatment, and the correlations were generally weaker than for the high CU microbiome mice (Figure 5d). Collectively, these results corroberate our previous assertion that host metabolic health outcomes were related to changes in microbiota composition in the high CU microbiome mice but not in the low CU microbiome mice. Furthermore, these results indicate that the microbiota compositional changes contributing to the negative metabolic effects of high CU microbiome mice consuming an extruded diet were due to changes in only a small number of ASVs, primarily from the *Lachnospiraceae* and *Ruminococcaceae* families.

### ASVs associated with extrusion were linked with elevated sugar degradation and SCFA production in the high CU microbiome mice

We next hypothesized that ASVs associated with consuming either extruded diet may be contributing increased functionality related to microbiome energy harvest from the diet and thus causing negative metabolic responses in the mice. We therefore examined the predicted functions contributed by ASVs associated with extrusion versus those associated with the whole wheat porridge diet. We focused our analysis on metabolic pathways related to degradation and energy harvest from undigested components of the dietary treatments.

**Figure 6.**
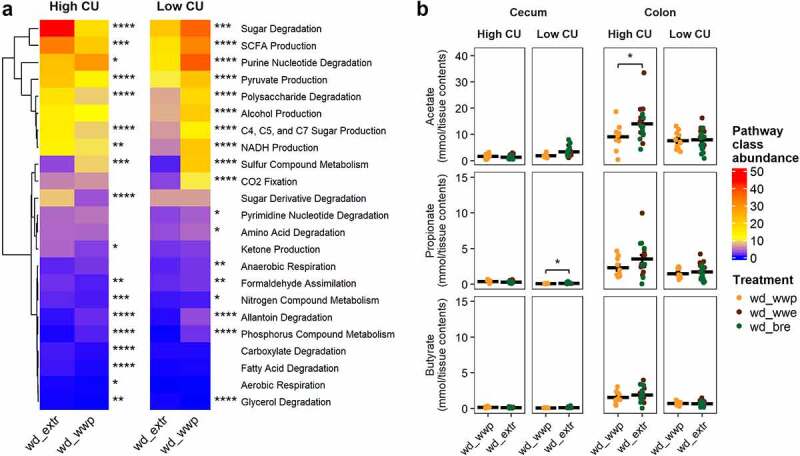
**ASVs associated with extrusion were predicted to contribute to elevated sugar degradation and SCFA production in the high CU microbiome mice**. Predicted (PICRUSt2) metabolic pathway classes with significant differences between extruded and non-extruded diets (a); SCFA pools in the cecum and colon measured at necropsy (b); wd_extr, extruded diets (wd_wwe and wd_bre); *p < .05, **p < .01, ***p < .001, ****p < .0001 (Wilcoxon-test with Benjamini-Hochberg p-value adjustment).

In the high CU microbiome mice, metabolic pathways associated with sugar fermentation and SCFA were the most abundant, with significantly more sugar degradation and SCFA production contributed by ASVs associated with extruded diets compared with diets that were not extruded (Figure 6a). (Note that ‘Sugar Degradation’ was a separate category from ‘Polysaccharide Degradation,’ which was also higher for ASVs associated with the extruded diets but of lower abundance than ‘Sugar Degradation.’)

As with the high CU microbiome mice, sugar degradation and SCFA production were the most abundant metabolic pathways associated with differentially abundant ASVs in the low CU microbiome mice (Figure 6a). However, the magnitude of difference between the extruded and non-extruded diets was smaller than that for the high CU microbiome mice. Also, these pathways were elevated in the ASVs associated with the non-extruded whole wheat porridge diet rather than the extruded diets.

Because the differential ASVs associated with extrusion versus porridge were predicted to contribute primarily to sugar degradation and subsequent SCFA production, we examined the SCFA concentrations in the cecal and colonic contents of mice. A slight, but significant, increase in propionate production was found in the ceca of mice consuming the extruded diets compared with the whole wheat porridge diet in the low CU microbiome mice (Figure 6b). However, overall SCFA production in the cecum was very low for all treatments. Much greater SCFA production was found in the colon, where the high CU microbiome mice consuming the extruded diets had significantly higher colonic acetate production. These results suggest that the ASVs contributing to the negative effects of extrusion on host health were involved in increased energy harvest due to excessive acetate production.

### ASVs associated with extrusion were different during an in vivo study compared with prior in vitro study but showed similar predicted functionality

Notably, the ASVs associated with the extruded and non-extruded diets in this *in vivo* study were not the same as those associated with the whole wheat extrudate and whole wheat porridge in the prior *in vitro* study. As mentioned, *in vitro* fermentations with the whole wheat extrudate resulted in increases in ASVs from *Acinetobacter, Enterococcus*, and *Staphylococcaceae* at the expense of key cell wall degrading bacteria from *Ruminococcaceae* and *Lachnospiraceae in vitro* (Figure 1e). In contrast, in the present study, the extruded diets were associated with ASVs from *Lachnospiraceae* and *Ruminococcaceae*, and the whole wheat porridge resulted in elevated ASVs from several other families, including those from phyla other than Firmicutes.

**Figure 7.**
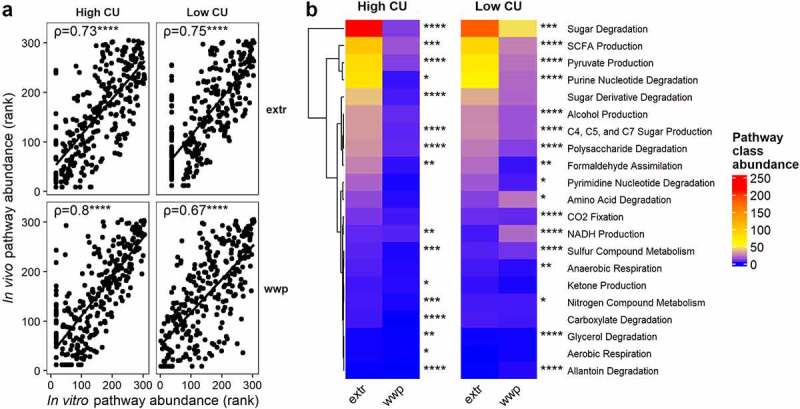
**ASVs associated with extrusion were different during an *in vivo* study compared with prior *in vitro* study but showed similar predicted functionality**. Spearman’s rank correlation plots between predicted pathway abundances contributed by differentially abundant ASVs *in vivo* versus *in vitro* in high and low carbohydrate utilization microbiomes on extruded (extr) or non-extruded (wwp) substrates (a); mean abundances of pathway classes with significant differences between extr and wwp (b); *p < .05; **p < .01; ***p < .001; ****p < .0001 (Wilcoxon-test with Benjamini-Hochberg p-value adjustment).

Despite this inconsistency, we speculated that ASVs enriched *in vivo* and *in vitro*, although different, may perform similar functions under each dietary treatment. We therefore examined the predicted function of ASVs previously associated with extrusion and porridge (non-extruded) from our *in vitro* study in the same manner as was done for this *in vivo* study. The predicted functions of the differential ASVs between the present *in vivo* and the previous *in vitro* study were indeed highly correlated (Figure 7a), indicating that these ASVs may be performing similar functions under different experimental conditions. Also similar to the present study, sugar degradation and SCFA production functions were significantly elevated by ASVs associated with extrusion in the high CU microbiome mice *in vitro* (Figure 7b). This difference was also observed in the low CU microbiome mice, although the magnitude of difference between the extruded and porridge samples was smaller. This finding differs from the present *in vivo* study, where the ASVs associated with porridge diets were predicted to contribute elevated sugar degradation and SCFA production compared with the ASVs associated with the extruded diets. Together, these results support the conclusion that the functions performed by differentially abundant ASVs among different dietary treatments are similar between our *in vitro* and *in vivo* studies despite unique changes in microbiota composition under these two different experimental conditions. Furthermore, the host metabolic outcomes observed during the *in vivo* study were predictable based on the projected functions obtained from the *in vitro* study.

## Discussion

In the present study, human microbiota-associated mice inoculated with one of two microbiomes that varied in their *in vitro* CU capability (high versus low) were fed processed whole grain diets for 12 weeks. Mice consuming either extruded diet (whole wheat or brown rice) were less metabolically healthy than mice consuming the other diets regardless of microbiome; however, the negative effects of consuming the extruded diet were exacerbated in mice harboring the high CU microbiome compared with the low CU microbiome. Because similar host metabolic responses to consuming an extruded diet were seen in both microbiomes – except for an exaggerated effect in the high CU microbiome mice – we suggest that the negative effects of extrusion may involve both microbiome-independent and -dependent mechanisms. First, extrusion can have a microbiome-independent influence on glucose metabolism, whereby it increases the glycemic index and load of cereals and whole grains by making the starch more rapidly available and digestible.^43–45^ This is supported by our data showing that the extruded samples had a much higher proportion of gelatinized starch, which is known to digest more rapidly than ungelatinized starch,^46^ compared with the whole wheat porridge sample. The change in glycemic index for a particular grain processed via extrusion or not has been shown to be much greater than the difference between grains processed using the same conditions,^47^ further suggesting a dramatic influence of processing on the glycemic index of whole grains that exceed the differences in composition among grain types. This scenario is particularly true when extrusion conditions are extreme^48^ as they were in this study. Consequently, the negative effects of the extruded diets in both microbiome backgrounds could be due, in part, to the increased glycemic index of the extruded diets.

Nevertheless, high CU microbiome mice clearly experienced exaggerated negative effects when consuming extruded diets. These exacerbated negative effects were likely mediated via microbiome-dependent pathways because they were not observed in the low CU microbiome mice and because the high CU microbiome mice consuming the extruded diets clustered together for both changes in metabolic health and microbiota composition during feeding. Based on our results showing that ASVs associated with the extruded diets contributed to elevated sugar degradation and SCFA production, together with elevated colonic acetate production in mice fed the extruded diets, we infer that the microbiome-dependent effects of extrusion on host health were related to increased energy recovery brought about by increased fermentation of carbohydrates and acetate production by the microbiome.

Although SCFA are beneficial products of fermentation of dietary fibers by the microbiome,^49^ they also provide a means of energy recovery (increased Calories) from the diet.^50^ Acetate in particular has generated a fair amount of controversial literature about its benefits versus detrimental effects on host metabolic health.^51–54^ Acetate arising from gut microbiota metabolism is absorbed by colonocytes and transported to the liver where a portion of it is available for lipogenesis and cholesterol synthesis.^55^ However, most microbiota-generated acetate exits the liver and enters peripheral circulation for uptake by adipose and muscle tissue^55^ where it is then either oxidized to produce ATP or used as a substrate for *de novo* lipogenesis depending on energy needs.^50^ Alternatively, circulating acetate can bind to G-protein coupled receptors and precipitate host metabolic benefits by reducing appetite and increasing both insulin sensitivity and energy expenditure.^56^ Therefore, under normal circumstances, any energy recovery contributed by acetate arising from gut microbial fermentation of dietary fiber fermentation is likely counteracted by reduced appetite and increased energy expenditure, thus ultimately leading to health benefits for the host. However, under *ad libitum* consumption of an energy dense diet, the positive effects of acetate may not outweigh the additional energy it supplies. For example, Perry et al.^57^ showed that mice consuming a high fat diet (60% kcal from fat containing 6.5% dietary fiber as cellulose) produced elevated levels of acetate, which were responsible for increased insulin resistance and body weight gain. Additionally, human fecal samples from obese individuals are repeatedly reported to have higher acetate concentrations compared with those obtained from normal weight individuals,^58–61^ even though people with obesity tend to eat lower fiber diets.^62^ In our study, although all diets contained equivalent concentrations of dietary fiber, the extruded diet had a portion of the cellulose replaced with dietary fibers that were easily fermentable for mice with the high CU microbiome. Replacement of poorly fermentable dietary fibers (i.e., cellulose or, to a lesser extent, the dietary fibers in the whole wheat porridge), with the available fermentable fibers in the extruded diets likely led to increased acetate production and energy harvest from the diet and subsequent negative health effects for the mice.

Our observation of higher acetate concentrations in the colon versus the cecum of high CU mice is peculiar. The cecum is usually the primary site of dietary carbohydrate fermentation, and SCFA concentrations decrease along the colon due to absorption by host colonocytes.^63^ However, Arora et al. showed that cecal SCFA were dramatically reduced in mice consuming a defined diet containing 10% cellulose compared with mice consuming chow diets.^64^ When all of the cellulose was replaced with a fermentable flaxseed fiber, total cecal SCFA concentrations were partially restored but still significantly lower than mice on a chow diet. In our study, only a portion of the cellulose was replaced with the whole grains (0.3–1.8% of the diet or 5–30% of the cellulose). Perhaps the low concentration of fermentable fiber in our study was not sufficient to elicit substantial SCFA production in the cecum. Moreover, whole grain dietary fibers are known for their slow and incomplete fermentation properties,^65^ which may explain why fermentation of the fiber in our test diets was delayed into the colon.

Another potential explanation for the elevated acetate production in the colons of high CU mice consuming extruded diets may involve fermentation of other substrates such as host polysaccharides after the fermentable carbohydrates supplied by the extruded diets were exhausted. The observation that acetate was only elevated in the colons of high CU mice consuming the extruded whole grain diets, which were known to be more readily available to this microbiome,^9^ together with previous studies showing that whole grains tend to increase propionate or butyrate rather than acetate,^13,14,66,67^ support this explanation.

Remarkably, the method of grain processing (extruded versus porridge) had a greater effect on the metabolic health of the mice than the type of whole grain added to the diet (whole wheat versus brown rice). The extrusion processing utilized in this study was extremely severe, employing high temperature and screw speed and low moisture, while boiling grain into porridge is among the most mild treatments that can be used to process grain into an edible food product. Although we magnified the effects of processing by selecting very severe extrusion conditions and mild porridge conditions, we also did this for grain type, since brown rice is the lowest in dietary fiber among whole grains.^15^ Thus, our study highlights the importance of processing method on the interaction between whole grains and the gut microbiome. It should be noted, however, that the extrusion conditions utilized in this study were very severe and are not typically used in routine food production. Thus, our results probably represent a worst-case scenario for extrusion. Other more typical extrusion conditions may not elicit such a negative impact on host metabolic health.

In contrast to the extruded diet, the porridge diet did not result in negative health effects. Although we anticipated that porridge would provide significant metabolic improvements for the host compared to extrusion, we expected that it would also improve host health relative to the control Western diet. Despite tendencies for the whole wheat porridge diet to provide metabolic benefits over the control Western diet, the effects were not significant. As discussed previously, all our diets were fabricated with equivalent concentrations of dietary fiber – the control Western diet containing cellulose and the whole wheat porridge diet containing a portion of cellulose replaced with the dietary fibers from the porridge. However, because the diets were formulated with the processed whole grains rather than isolated cereal fibers, the dietary fibers from the porridge comprised only 1.8% of the overall diet. Other studies demonstrating beneficial metabolic benefits of dietary fiber have provided higher levels by incorporating bran into the diet and removing all of the cellulose ingredient (5.8% fiber from bran).^68^ Although our approach was chosen to approximate the recommended intake of three servings of whole grain per day for Americans,^6^ the level of inclusion of these fibers in the diet was perhaps still too low to elicit significant positive effects on host metabolic health.

Finally, we found discrepancies between taxa associated with extruded and non-extruded diets in this *in vivo* study compared with our previous *in vitro* study. Specifically, extrusion was associated with decreases in some *Lachnospiraceae* and *Ruminococcaceae in vitro*, but several ASVs from these families were elevated with extrusion *in vivo*. This differential result is likely due to 1) imperfect establishment of human gut microbes into a mouse model^69^ and 2) consumption of other dietary components (in addition to the processed whole grains) that were not included in the *in vitro* study but likely influenced microbiota composition during this *in vivo* study. However, the predicted metabolic pathways contributed by ASVs associated with the extruded or non-extruded diets were highly correlated between the *in vivo* and *in vitro* results, suggesting that although the ASVs were different, they were performing similar functions. Importantly, the metabolic phenotypes observed in the present study were entirely predictable based on the results from the *in vitro* study.

## Conclusions

The present study provides novel insight into the influence of whole grain processing method on host metabolic health. Surprisingly, the impact of processing was greater than the effect of grain type, where extruded whole wheat and brown rice both worsened host metabolic health compared to whole wheat porridge. This outcome was observed independently of the microbiome’s CU capacity; however, mice harboring the high CU microbiome exhibited an exaggerated host metabolic response to food processing, where severe extrusion resulted in a greater number of significant negative effects on host health than mice harboring the low CU microbiome. Thus, the negative effects of extrusion on host health were attributed to microbiome-independent effects in both microbiomes – likely an increase in glycemic index of the diet – and microbiome-dependent effects in only the high CU microbiome mice. In mice carrying the high CU microbiome, grain processing had the greatest effects on the abundance of *Lachnospiraceae* and *Ruminococcaceae*, which contributed to elevated sugar degradation and acetate production by the microbiome when feeding extruded grains versus porridge, thus resulting in increased energy recovery. We encourage funding agencies and the food industry to consider with greater urgency the importance of developing food processing strategies that target the microbiome and decrease the public health crises caused by poor diet.^70^

## Supplementary Material

Supplemental Material

## Data Availability

The datasets supporting the conclusions of this article are included within the article and its additional files. Additional files include: 1) metadata table, 2) OTU table 3) taxonomy table, 4) tree file, 5) biological sequence file, 6) PICRUSt2 pathway abundance contribution table, 7) Metacyc pathway class descriptions, 8) mouse metabolic and SCFA data. Raw sequencing data (paired end reads in FASTQ) and metadata are available at the NCBI SRA: PRJNA690589.
