# Supplementary material for: Carbohydrate utilization by the gut microbiome determines host health responsiveness to whole grain type and processing methods
Source: Gut Microbes. 2022 Sep 21;14(1):2126275. doi: 10.1080/19490976.2022.2126275 (PMC9519025; doi:10.1080/19490976.2022.2126275)
Supplement: Supplemental Material [file KGMI_A_2126275_SM9231.zip › Big MAC v17c_suppl.docx]

**Table S1. Composition of processed whole grain samples.^A^**

| Component | Whole wheat porridge (wwp) | Whole wheat extrudate (wwe) | Brown rice extrudate (bre) |
| --- | --- | --- | --- |
| Dietary fiber | 9.70 ± 0.21 a | 8.75 ± 0.07 b | 1.69 ± 0.01 c |
| Starch | 66.5 ± 2.6 b | 58.2 ± 2.9 c | 76.3 ± 5.8 a |
| Degree of gelatinization  (% of starch) | 68.3 ± 4.1 c | 95.7 ± 3.5 b | 88.6 ± 3.1 b |
| Fructose | 0 ± 0 b | 0.037 ± 0.015 a | 0 ± 0 b |
| Glucose | 0.032 ± 0 c | 0.055 ± 0.006 b | 0.065 ± 0.002 a |
| Sucrose | 0.645 ± 0.015 c | 0.711 ± 0.017 b | 0.948 ± 0.037 a |
| Protein | 15.5 ± 0.1 a | 13.9 ± 0 b | 8.58 ± 0.04 c |
| Lipid | 1.1 ± 0.23 | 0.782 ± 0.079 | 0.656 ± 0.028 |

^A^ Results are expressed as percent dry basis except where indicated otherwise; mean ± standard deviation; n=2 (dietary fiber, protein, lipid); n=3 (degree of starch gelatinization, fructose, glucose, sucrose); n=4 (starch); means followed by difference lower-case letters are significantly different within row (Tukey’s HSD p<0.05).

**Table S2. Formulation and composition of mouse diets.**

| Component | lf | wd | wd_wwp | wd_wwe | wd_bre |
| --- | --- | --- | --- | --- | --- |
| Formulation*, g/kg |  |  |  |  |  |
| Casein | 190 | 233 | 202 | 199 | 209 |
| L-Cystine | 2.8 | 3.5 | 3.4 | 3.4 | 3.4 |
| Corn Starch | 521 | 85 |  |  |  |
| Maltodextrin 10 | 142 | 117 | 77 | 82 | 67 |
| Sucrose |  | 201 | 198 | 196 | 193 |
| Whole Wheat Porridge |  |  | 185 |  |  |
| Whole Wheat Extrudate |  |  |  | 185 |  |
| Brown Rice Extrudate |  |  |  |  | 185 |
| Cellulose | 47 | 58 | 39 | 42 | 53 |
| Lard | 19 | 207 | 204 | 201 | 199 |
| Soybean Oil | 24 | 29 | 27 | 27 | 27 |
| Mineral Mix S10026 | 9.5 | 12 | 11 | 11 | 11 |
| Dicalcium Phosphate | 12 | 15 | 15 | 15 | 15 |
| Calcium Carbonate | 5.2 | 6.4 | 6.3 | 6.2 | 6.2 |
| Potassium Citrate, 1 H2O | 16 | 19 | 19 | 19 | 18 |
| Vitamin Mix V10001 | 9 | 12 | 11 | 11 | 11 |
| Choline Bitartrate | 2 | 2 | 2 | 2 | 2 |
| Red Dye #40, FD&C | 0.024 | 0.058 |  |  | 0.028 |
| Blue Dye #1, FD&C | 0.024 |  | 0.057 | 0.028 |  |
| Yellow Dye #5, FD&C |  |  |  | 0.028 | 0.028 |
| Nutrients, kcal% |  |  |  |  |  |
| Protein | 18 | 18 | 18 | 18 | 18 |
| Carbohydrate | 71 | 36 | 36 | 36 | 36 |
| Fat | 11 | 46 | 46 | 46 | 46 |
| Dietary fiber, g/kg |  |  |  |  |  |
| Whole diet | 47 | 58 | 57 | 57 | 56 |
| From processed grain | 0 | 0 | 18 | 15 | 3 |
| From cellulose ingredient | 47 | 58 | 39 | 42 | 53 |

* Casein, corn starch, maltodextrin, sucrose, lard, soybean oil, and cellulose were reduced in the grain-containing samples compared with the control Western diet to keep the macronutrient composition consistent for total protein, starch, fat, and dietary fiber among each diet.

**
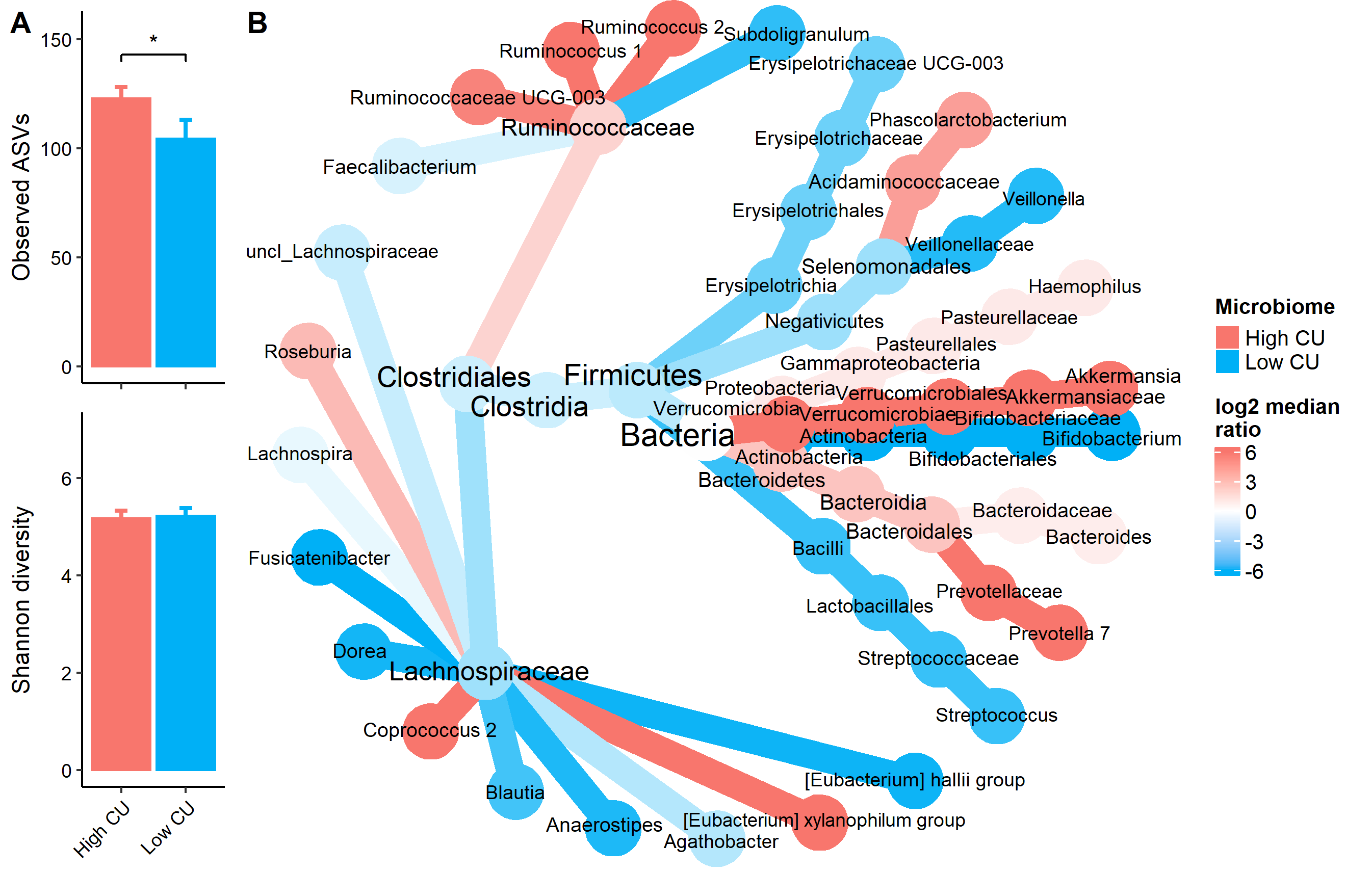
Fig. S1. Microbiome composition of fecal donors distinguished high versus low carbohydrate utilizing microbiomes.** Fecal donor α-diversity (A) and composition (B) of the fecal microbiomes; subpanel B shows only the top 25 most abundant genera and log_2_ median ratios of relative abundances; CU, carbohydrate utilization; *p<0.05, ****p<0.0001 (t-test).


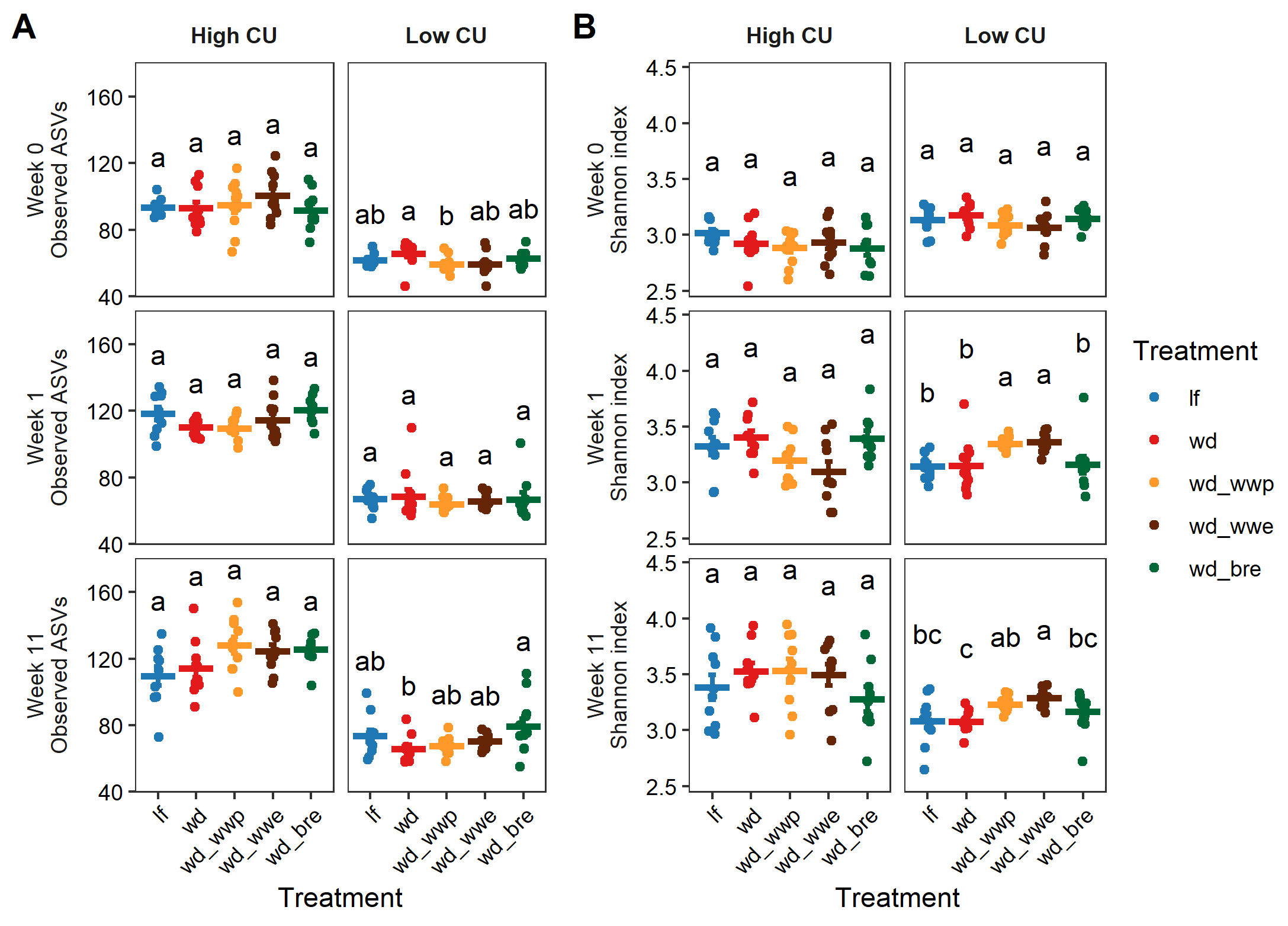


**Fig. S2. α-Diversity of mice inoculated with high carbohydrate utilization (CU) or low CU microbiomes after 12 weeks of feeding.** Observed ASVs (A), Shannon diversity (B); lf, low fat control; wd, Western diet; wd_wwp, wd + whole wheat porridge; wd_wwe, wd + whole wheat extrudate; wd_bre, wd + brown rice extrudate; treatments marked with different letters within panel (abc) are significantly different from one another (Dunn test p<0.05).
